# Psychiatrists' understanding and use of psychological formulation: a qualitative exploration^†^

**DOI:** 10.1192/pb.bp.115.051342

**Published:** 2016-08

**Authors:** Roxanna Mohtashemi, John Stevens, Paul G. Jackson, Stephen Weatherhead

**Affiliations:** 1 Lancashire Care NHS Foundation Trust and Lancaster University; 2 Mersey Care NHS Trust

## Abstract

**Aims and method** To establish an initial conceptualisation of how psychiatrists understand and use formulation within adult psychiatry practice. Twelve psychiatrists took part in semi-structured interviews. Transcripts were analysed using a constructivist grounded theory methodology.

**Results** Formulation was conceptualised as an addition to diagnosis, triggered by risk, complexity and a need for an enhanced understanding. Participants valued collaborative formulation with psychologists. Multiple contextual factors were perceived to either facilitate or inhibit the process. Barriers to formulation led to a disjointed way of working.

**Clinical implications** Findings contribute to an understanding of formulation within psychiatry training and practice.

Psychiatrists' training criteria require them to demonstrate ‘the ability to construct formulations of patients’ problems that include appropriate differential diagnoses'^1^ and a ‘careful clinical history and concise summary of the social, psychological and biological factors that may have contributed to developing a given “mental disorder”’.^2^ Despite such guidance, a survey asking psychiatrists to list what should be included in a diagnostic formulation revealed a lack of consensus among participants.^3^ A similar finding was reported in a survey for examiners of the Membership of the Royal College of Psychiatrists (MRCPsych) clinical exams.^4^ However, these studies are now dated and there have been no similar studies published since that may clarify whether there is still a lack of consensus on what a formulation should comprise and whether a psychological understanding is used at all. This has become particularly relevant given recent debates on the relative utility of psychiatric diagnosis versus psychological formulation,^5^ with the latter defined as ‘a hypothesis about a person's difficulties, which links theory with practice and guides the intervention’.^6^ The formulation should be based on psychological principles and be tentative and open to revision.^6^

Individual case formulations are traditionally incorporated in some form into the majority of psychotherapeutic modalities^7–9^ and developed as a ‘recursive process of suggestion, discussion, reflection, feedback and revision’ during therapy.^10^ Research into the impact of formulation during therapy suggests that it may increase levels of hope and understanding in patients.^11,12^ Additional research into the use of formulation is warranted, particularly given the relative lack of participation representation from psychiatrists within empirical studies focused on team-based formulation in mental health settings.^13–15^

## Rationale for the current study

Existing research does not offer an understanding of how psychiatrists specifically understand formulation and whether they value team formulation and/or consultation with psychologists to develop a psychological understanding of their patients' difficulties. To enhance clinical practice for both staff and patients it is necessary to have a clear understanding of what is understood by the concept of formulation and how it is used. This is in keeping with recommendations to continue to share a dialogue with other disciplines within the field of mental health practice.^6^

## Method

A constructivist grounded theory approach^16^ was adopted to explore three questions:

1. How do psychiatrists understand formulation?
2. How do psychiatrists use formulation in their everyday practice?
3. Do psychiatrists value the process of formulation with psychologists and/or in a team?

Ethical approval was obtained from Lancaster University Research Ethics Committee and National Health Service (NHS) research and development approval was obtained from the relevant NHS trust prior to conducting the research. Twelve participants working in different services across four NHS trusts were recruited. All participants had experience of formulation within adult services. They were aged between 33 and 67 years and ethnicity was diverse. All participants chose a pseudonym to ensure anonymity. Data were collected via one-to-one semi-structured interviews using an interview schedule developed with the support of the psychiatrist supervising the project. All interviews were audio recorded.

Analysis was informed by constructivist grounded theory, acknowledging ‘truth’ as socially constructed through language and social interactions.^16^ Data were analysed inductively, beginning with line-by-line coding, through to constant comparison of developing codes and concluding with multi-level relational analysis of emergent codes.^17^ Due to the pragmatic nature of carrying out inductive research as part of a time-limited professional doctorate, ‘theoretical sufficiency’ and related sampling strategies^18^ rather than ‘saturation’ were established as an initial conceptualisation of participants' experiences of the research topic. ‘Sufficiency’ was considered when conceptual categories did not require revision in light of fresh data. This is in contrast to ‘data saturation’, which is achieved when interviews no longer offer new insights.^19^

### Reflexivity

It was important to remain reflexive^19^ throughout the research process in order to be aware of possible biases towards the data and emerging theory. Regular supervision provided by an experienced academic clinician facilitated reflection throughout, from conceptualisation to completion. The researcher also discussed the emerging theory and diagrammatic representation with an additional research tutor who was well versed in grounded theory, which enabled the identification of gaps and informed the interview schedule for the final three interviews, for example finding out more about the dichotomy between using formulation and not needing formulation.

## Results

**Fig. 1:**
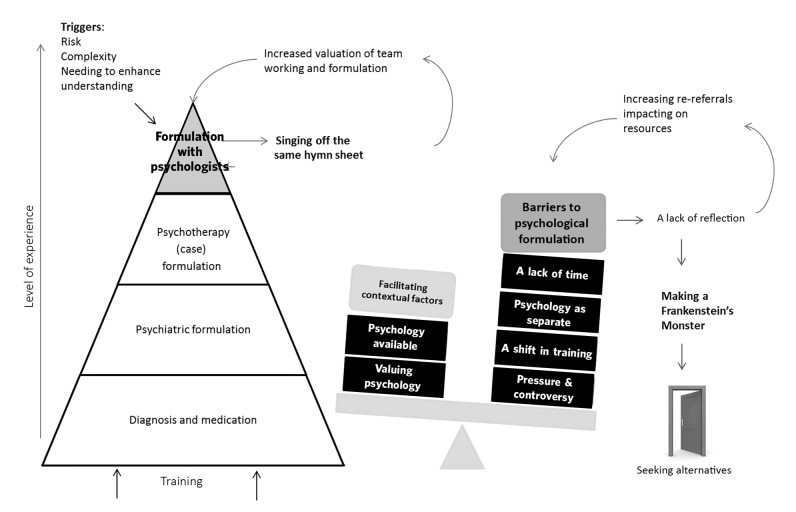
Model representing psychiatrists' understanding and use of psychological formulation.

The analysis identified 111 focused codes which were grouped and re-grouped into 4 conceptual categories of: (1) conceptualising formulation; (2) singing off the same hymn sheet; (3) barriers to formulation; and (4) making a Frankenstein's monster. These are presented in narrative form below, together with supporting quotes taken from the original data. A diagrammatic representation of the findings can be seen in Fig. 1.

### Conceptualising formulation

Participants' understanding of formulation was described as a developmental process, contingent on psychiatry training and clinical practice. Diagnosis was noted to be the foundation of their role, prioritised alongside medication. A psychological understanding was not always perceived as necessary; patients' difficulties were sometimes understood to be purely biological in nature: ‘if someone is bipolar, it's bipolar, you know they're manic, you don't need to [formulate] … you do [a] diagnosis’ (Stephanie). Dave explained how individual differences contributed to whether or not psychiatrists used formulation:

‘I think it depends on the psychiatric background of the person … I have an analytical background, so it's much more easy for me to do the formulation but if you ask me … whether it's being done regularly by all the consultants, or medics, I would say unlikely.’

Overall, diagnosis and medication were the main foci of participants' perceived roles and a psychological understanding was not always considered necessary.

Formulation was described as a heuristic device to enhance understanding, which led to a number of different outcomes including the offering of hope, informing reports, improving medication concordance, signposting to treatment and management of care. A diagnosis was not perceived to be sufficient in this respect, as Jack described:

‘I might see a patient who was obsessional in nature … I can give them a DSM diagnosis … but actually I'd quite like to know where has that [sic] come from … that helps me understand the behaviour, as it does in my opinion the patient. Because giving the patient a label and a diagnosis is all very well, but helping them understand where it's come from is, I think, that's part of the hope bit.’

Complexity, a high level of risk and patients who accessed services frequently were perceived as reasons by participants to warrant either referral to psychology or consultation with psychologists. For example, patients within Stephanie's service who self-harmed, as well as those who were ‘in and out of hospital’, were prioritised for psychological support. This related to Jack's and Rob's observation of having more psychology presence in forensic services, where there was a greater emphasis on risk.

### Singing off the same hymn sheet

Some participants described entering a process of creating a unified understanding between psychologists and psychiatrists, resulting in the successful integration of different epistemological positions. This process was named by Jack as ‘singing off the same hymn sheet’ and dependent on a number of contextual factors.

Facilitating contextual factors included availability of psychologists, positive working relationships and an expressed individual interest in psychology by participants. A positive relationship with psychologists available not just within the service, but with those who were in close physical proximity and available to consult with when needed was an important requirement for being able to enter into a process of integration:

‘ … the conversations with her [the psychologist], just kind of developed into thinking a bit more … we had an interesting meeting with him [the patient], me and the psychologist and the nursing staff and then afterwards we were able to have a ten minute conversation, the psychologist and I, about what we felt we got out of that’ (Jane).

A joint understanding enabled the difficult dynamics between staff and patient to be explored with the staff team. Formulation was also used as a language to communicate with team members, professionals outside the service and patients themselves: ‘I had to get forensic commissioners on board … and speak to forensic colleagues … being able to take it back to a basic formulation really helped’ (Jane).

Psychologists were described as playing a key role as part of the team process:

‘We use it with the psychology team … we do a formulation at the end to bring all that information together and say, well, where is this person likely to be going on their psychological journey and how can we prevent things from happening or understand why they're happening. And that informs the multidisciplinary team’ (Jack).

Participants expressed a desire to see an increase in psychologists training nursing staff in basic formulation skills. Rebecca emphasised the need to ‘maximise the effects of individual therapy’ by equipping care coordinators with psychological formulation and intervention skills: ‘I think for me whats more important is to actually build skills within the team so that practitioners would be able to have some basic skills around formulation’ (Rebecca). All but one of the participants felt it was a misuse of resources for psychologists to spend the majority of their time delivering therapy to a relatively small number of individuals, rather than consulting with the team.

### Barriers to formulation

Participants' understanding and use of formulation was perceived to be dependent on the wider system. This was reflected in interviews where a large amount of time was spent talking about different contexts, for example the politics surrounding psychiatry and limitations within NHS services. Participants perceived themselves to be faced with multiple barriers that affected their ability to formulate and think reflectively.

The allocation of 15 min for clinical appointments was described as insufficient to be able to use formulation directly with the patient, resulting in a ‘robotic’ and risk-focused approach (Dalglish). Perhaps because of a lack of time, participants spoke more about formulation being used to enhance their own understanding rather than directly enhancing the patient's understanding. Participants described being under immense pressure to make quick decisions within their roles, which did not allow for formulating or reflective practice. Anita noted ‘we don't think; we just do', while Dave commented: ‘they say you have to do reflective practice … we don't have the time’.

In cases where a patient was admitted to a ward on a short-term basis, participants thought the time taken to create a formulation was longer than the patient admission length. Jane described the process in a psychiatric intensive care unit (PICU): ‘you're trying to gather information from a variety of sources with someone you've not met before, who might not be with you for more than a few weeks so that takes a lot of time’.

Some participants conceived a pressure to conform to the medical model, needing to use diagnosis as a priority, to provide medication and classification. This pressure came from multiple stakeholders, from the patient to the general practitioner (GP): ‘Some people really want to be medicalised’ (Rob) and ‘The GP wants more a medical model. He just wants a number’ (Vivek).

Psychology was seen by some participants as a threat or attack to the profession of psychiatry, with some psychologists being described as being ‘anti-psychiatry’. This was a barrier within the workplace as psychologists perceived as anti-psychiatry were thought to behave in a defensive way in their interactions with psychiatrists.

Participants described professional rivalry causing psychiatrists to revert to a dominantly biological understanding of distress:

‘ … there's [sic] some people who are reacting against that [challenging diagnosis] who are seeking to define what they do and in some ways narrow their understanding of a reductionist model to a purely biological, chemical based model’ (Rob).

Overall, the majority of participants perceived professional rivalry as unhelpful and wanted to see a move towards a process of acceptance and integration of psychologists and psychiatrists.

### Making a Frankenstein's monster

The consequences of not being able to develop a psychological understanding owing to one or more of the barriers described above led to a perceived lack of reflection. This resulted in participants resorting to a number of alternative approaches.

There was a perceived overreliance on a medical understanding of distress, as a consequence of limited resources. For example, Dave reflected that ‘you might end up prescribing medication because you might have to come across as doing something. But you know that it's [the illness is] psychological’. This was described by Michael as a ‘top-down’ pressure to conform to using medication, whereas Dave perceived the pressure also coming from patients themselves: ‘some of the time the patient comes in and says well I can't be bothered to sit down and talk so could you give me a pill’.

Zadok described a process of treating ‘complexity’ with multiple types of medication with a consequence of not knowing what had worked. He described trying to understand a patient presenting with information perceived as incoherent: ‘he's got some sort of impulsivity, [I have] given him some SSRI's [selective serotonin reuptake inhibitors] for that, and on the other hand he doesn't get on very well with his mother and it doesn't really fit together’. Overall, the combination of a lack of integrated understanding of a patient, overreliance on medication and general lack of resources was described as creating a ‘Frankenstein's monster’ (Zadok), where the monster represented the process of disjointed practice by psychiatrists.

The approach maintained barriers to understanding and using psychological formulation, resulting in patients being treated without a holistic understanding, which meant that patients kept returning to the service: ‘It's a false economy in my view … because you haven't done the formulation you don't understand what's going on … you just make more work for yourself’ (Rebecca).

Three participants described dissatisfaction at working in a pressurised environment, which eventually led them to ‘seek alternatives’ such as entering academia, working privately or moving into other specialisms. For example, Michael talked about moving from adult services into child services, as he perceived there to be less pressure to prescribe medication and more time to think psychologically.

## Discussion

Findings from this study offer an initial conceptualisation of a sample of psychiatrists' understanding and use of formulation, highlighting how utilisation was dependent on experiences within both psychiatry training and clinical practice. Previous research into professionals' views of formulation comprises small-scale evaluations of staff experiences of team formulation,^14^ psychologists' perceived roles in creating and sharing formulations within multidisciplinary teams,^20^ and numerous opinion pieces promoting the use of formulation either alongside^21^ or as a replacement to diagnosis.^10^

Despite conflicting theoretical perspectives between psychologists and psychiatrists, participants reported an integration of these understandings while ‘singing off the same hymn sheet’. This involved clearly defined roles, working collaboratively and having space to ‘think’ together to develop a shared understanding.

The more psychiatrists are exposed to the benefits of team formulation and working collaboratively with psychologists, the more they integrate that into their everyday practice. This mirrors findings from a survey over 30 years ago where consultant psychiatrists were more likely to incorporate a psychological understanding into a diagnostic formulation than junior psychiatrists.^3^ Not only does this emphasise the benefits of professional practice gained from multidisciplinary team-working, it suggests a gap in psychiatry training for team formulation.

The lack of time, emphasis on risk and format of psychotherapy training seemed to lead to formulation being viewed as a discrete ‘event’^6^ or one-off activity which may have been perceived by some participants as potentially lengthy and an ‘inessential luxury’. However, psychological formulation is arguably a more holistic and longitudinal process, which could be considered an intervention in itself.

### Clinical implications

There is a need for the Royal College of Psychiatrists to recognise the role of psychologists in promoting psychological thinking across teams.^21^ It may be beneficial to reconsider how formulation is conceptualised to psychiatry trainees, perhaps incorporating psychological ways of thinking that are outside of the traditional one-to-one psychotherapy format. Teaching from psychologists alongside psychiatrists during training for both disciplines may cultivate an integrated way of working from the outset, facilitating cross-disciplinary working in clinical practice.^22^

Psychiatrists should have access to supported and reflective practice throughout their continued development.^1^ The process of team formulation, facilitated by a psychologist, offers a ‘thinking space’ for staff teams to reflect and formulate. This is in keeping with guidelines promoting psychologists to integrate their work into teams while maintaining their ‘unique identity and contribution (e.g. offering a constructive counter-balance to the ‘medical model’).^23(p.3)^ Given the indicated barriers to collaborative formulation, it may be more appropriate for psychologists to work informally, supporting the team and ‘chipping in’ with psychological thinking.^20^ While acknowledging no simple solution, examples of initiatives of positive practice are outlined in the British Psychological Society document *Working Psychologically in Teams*.^23^

### Limitations

It is acknowledged that the researcher's background in clinical psychology, where the context is paramount to understanding people's difficulties, will have influenced the focus of the data on context and on formulation with psychologists. In response, the researcher used a number of different strategies to maintain neutrality, as discussed in Method. There may also have been some bias in that participants who responded to the invitation to participate in the study may have felt more strongly about the research topic than the profession in general. Indeed, many expressed a special interest in psychology, while others seemed to have strong opinions on the debate around formulation and diagnosis.

Findings cannot be wholly generalised to other settings, although the developed model did suggest ‘internal consistency’^24(p.91)^ owing to interaction of codes between conceptual categories. This indicates robustness of the model and potential to transfer the findings to psychiatrists working in similar settings, which is worth exploring in future research.

### Future research

Additional research is necessary to identify whether the findings from this study can be generalized to other settings such as primary care. Research may seek to clarify whether team formulation is a cost-effective endeavour, focusing on outcomes such as recovery or reduced use of medication.^25^ Furthermore, research focusing on whether formulation enhances the doctor-patient relationship may or may not highlight the need to think psychologically within time-limited appointments.

The use of formulation is triggered by risk or complexity and its goal is to enhance understanding. Contextual factors may influence the possibility for psychiatrists using formulation during their clinics and as part of a multidisciplinary team.

It is hoped our findings will contribute to a clearer definition of formulation within psychiatry training and practice. The need to maintain an open dialogue across disciplines is paramount in creating a holistic and integrated health service provision.

## References

[1] Royal College of Psychiatrists Good Psychiatric Practice: Continuing Professional Development. Royal College of Psychiatrists, 2010.

[2] American Psychiatric Association Diagnostic and Statistical Manual of Mental Disorders, 5th edn (DSM-5). APA, 2013.

[3] Hollyman JA Hemsi L What do psychiatrists understand by formulation? Psychiatr Bull 1983; 7: 140–3.

[4] Hollyman JA Hemsi L What do examiners understand by formulation? Psychiatr Bull 1983; 7: 165–7.

[5] Bracken P Thomas P Timimi S Asen E Behr G Beuster C Psychiatry beyond the current paradigm. Br J Psychiatry 2012; 201: 430–4. 23209088 10.1192/bjp.bp.112.109447

[6] Division of Clinical Psychology Good Practice Guidelines on the Use of Psychological Formulation. BPS, 2011.

[7] Beck J Cognitive Therapy: Basics and Beyond. Guildford Press, 1995.

[8] Ryle A Cognitive Analytic Therapy: Developments in Theory and Practice. John Wiley & Sons, 1995.

[9] Malan D Individual Psychotherapy and the Science of Psychodynamics. Butterworth, 1979.

[10] Johnstone L Dallos R Formulation in Psychology and Psychotherapy: Making Sense of People's Problems. Routledge, 2013.

[11] Chadwick P Williams C Mackenzie J Impact of case formulation in cognitive behaviour therapy for psychosis. Behav Res Ther 2003; 41: 671–80. 12732375 10.1016/s0005-7967(02)00033-5

[12] Shine L Westacott M Reformulation in cognitive analytic therapy: effects on the working alliance and the client's perspective on change. Psychol Psychother Theory Res Practice 2010; 83: 161–77. 10.1348/147608309X471334 19793412

[13] Berry K Barrowclough C Wearden A A pilot study investigating the use of psychological formulations to modify psychiatric staff perceptions of service users with psychosis. Behav Cogn Psychother 2009; 37: 39–48. 19364406 10.1017/S1352465808005018

[14] Picken A Cogan N The experiences of clinicians using formulation in adult mental health: an interpretative phenomenological analysis. Clin Psy Forum 2012; 233: 37–40.

[15] Summers A Psychological formulations in psychiatric care: staff views on their impact. Psychiatr Bull 2006; 30: 341–3.

[16] Charmaz K Constructing Grounded Theory: A Practical Guide through Qualitative Analysis. Sage Publications, 2006.

[17] Glaser BG Strauss AL The Discovery of Grounded Theory: Strategies for Qualitative Research. Aldine Publications, 1967.

[18] Birks M Mills J Grounded Theory: A Practical Guide. Sage Publications, 2011.

[19] Yardley L Demonstrating validity in qualitative psychology. In Qualitative Psychology: A Practical Guide to Research Methods (ed Smith JA): 235–51. Sage, 2008.

[20] Craddock N Mynors-Wallis L Psychiatric diagnosis: imperfect and important. Br J Psychiatry 2014; 204: 93–5. 24493652 10.1192/bjp.bp.113.133090

[21] Christofides S Johnstone L Musa M ‘Chipping in': clinical psychologists’ descriptions of their use of formulation in multidisciplinary team working. Psychol Psychother Theory Res Pract 2012; 85: 424–35. 10.1111/j.2044-8341.2011.02041.x 23080531

[22] Department of Health Mental Health: New Ways of Working for Everyone: Developing and Sustaining a Capable and Flexible Workforce. Department of Health, 2007.

[23] British Psychological Society New Ways of Working for Applied Psychologists in Health and Social Care: Working Psychologically in Teams. BPS, 2007.

[24] Gasson S Rigor in grounded theory research: an interpretative perspective on generating theory from qualitative field studies. In Handbook for Information Systems Research (eds Whitman M Woszczynski A): 79–102. Idea Group Publishing, 2003.

[25] Hollingsworth P Johnstone L Team formulation: what are the staff views? Clin Psychol Forum 2014; 257: 28–34.

